# CHA_2_DS_2_ ‐VASc score and prior oral anticoagulant use on endovascular treatment for acute ischemic stroke

**DOI:** 10.1002/acn3.52217

**Published:** 2024-10-09

**Authors:** Yukihiro Imaoka, Nice Ren, Soshiro Ogata, Hirotoshi Imamura, Yasuyuki Kaku, Koichi Arimura, Shogo Watanabe, Eri Kiyoshige, Kunihiro Nishimura, Syoji Kobashi, Masafumi Ihara, Kenji Kamiyama, Masafumi Morimoto, Tsuyoshi Ohta, Hidenori Endo, Yuji Matsumaru, Nobuyuki Sakai, Takanari Kitazono, Shigeru Fujimoto, Kuniaki Ogasawara, Koji Iihara, Close The Gap‐Stroke, J‐ASPECT Study Collaborators

**Affiliations:** ^1^ Department of Stroke and Cardiovascular Disease Next Generation Medical Research National Cerebral and Cardiovascular Center Osaka Japan; ^2^ Department of Neurosurgery, Graduate School of Medical Sciences Kumamoto University Kumamoto Japan; ^3^ Department of Preventive Medicine and Epidemiology National Cerebral and Cardiovascular Center Osaka Japan; ^4^ Department of Neurosurgery National Cerebral and Cardiovascular Center Osaka Japan; ^5^ Deparment of Neurosurgery, Graduate School of Medical Sciences Kyushu University Fukuoka Fukuoka Japan; ^6^ Graduate School of Engineering University of Hyogo Himeji Hyogo Japan; ^7^ Department of Neurology National Cerebral and Cardiovascular Center Osaka Japan; ^8^ Department of Neurosurgery Nakamura Memorial Hospital Sapporo Hokkaido Japan; ^9^ Department of Neurosurgery Yokohama Shintoshi Neurosurgery Hospital Yokohama Kanagawa Japan; ^10^ Department of Neurosurgery Kobe City Medical Center General Hospital Kobe Hyogo Japan; ^11^ Department of Neurosurgery Tohoku University Graduate School of Medicine Sendai Miyagi Japan; ^12^ Deparment of Neurosurgery, Institute of Medicine University of Tsukuba Ibaraki Japan; ^13^ Deparment of Medicine and Clinical Science, Graduate School of Medical Sciences Kyushu University Fukuoka Fukuoka Japan; ^14^ Division of Neurology, Department of Medicine Jichi Medical University Tochigi Japan; ^15^ Deparment of Neurosurgery Iwate Medical University Morioka Iwate Japan

## Abstract

**Objective:**

We evaluated the effect of CHA_2_DS_2_‐VASc score and prior use of oral anticoagulants (OACs) on endovascular treatment (EVT) in patients with acute ischemic stroke and atrial fibrillation (AF).

**Methods:**

Patients with AF who received EVT in 353 centers in Japan (2018–2020) were included. The outcomes were symptomatic intracerebral hemorrhage (sICH), in‐hospital mortality, functional independence, and successful and complete reperfusion. The effects of CHA_2_DS_2_‐VASc score, its components, and prior use of OACs were assessed via a multiple logistic regression model.

**Results:**

Of the 6984 patients, 780 (11.2%) used warfarin and 1168 (16.7%) used direct oral anticoagulants (DOACs) before EVT. Based on the CHA_2_DS_2_‐VASc score, 6046 (86.6%) presented a high risk (≥2 for males and ≥3 for females) while 938 (13.4%) had intermediate to low risks. Higher CHA_2_DS_2_‐VASc scores were associated with increased sICH, in‐hospital mortality, and decreased functional independence, regardless of prior OACs. For patients with a high‐risk category, prior DOACs increased the odds of successful and complete reperfusion (adjusted odds ratio [95% confidence interval (CI)], 1.27 [1.00–1.61] and 1.30 [1.10–1.53]). For those with integrated intermediate to low risks, neither prior warfarin nor DOAC affected the outcomes. Regardless of total CHA_2_DS_2_‐VASc scores, patients with congestive heart failure or left ventricular dysfunction, hypertension, age >75 years, or female benefited similarly from prior DOAC use.

**Interpretation:**

Prior DOAC use for patients with high‐ and selected intermediate‐risk CHA_2_DS_2_‐VASc scores increased prevalence of successful and complete reperfusion. These findings may provide supplemental evidence to introduce preventive DOAC for patients with AF.

## Introduction

Appropriate oral anticoagulants (OAC) introduction reduces stroke risk in patients with atrial fibrillation (AF).^1^ The 2023 ACC/AHA/ACCP/HRS guideline for the diagnosis and management of AF recommends the use of the CHA_2_DS_2_‐VASc score as a validated clinical assessment tool to assess the annual risk of thromboembolic events.^2^ CHA_2_DS_2_‐VASc scores of ≥2 and ≥3 for male and female patients with AF, respectively, usually represent a high risk of stroke (~>2% per year) and necessitate OAC introduction.^2^, ^3^, ^4^ Scores of 1 for males and 2 for females represent an intermediate risk (1%–2% per year) and a dilemma for clinicians in balancing stroke and bleeding risks. While specific information on patients with a CHA_2_DS_s_‐VASc score of 1 has been accumulated over the last few years,^5^, ^6^ further evidence on the validity of the CHA_2_DS_s_‐VASc score and impact of OAC introduction is warranted.

In addition to its original purpose, the CHA_2_DS_2_‐VASc score is useful for predicting short‐ and long‐term outcomes of patients with ischemic stroke regardless of AF. The CHA_2_DS_2_‐VASc score predicts 3‐month mortality and functional outcomes, and 5‐year mortality after stroke in patients without AF.^7^, ^8^ The severity and outcomes of acute ischemic stroke (AIS) have improved within the past 20 years^9^ owing in part to the increasing use of direct oral anticoagulant agents (DOACs) for patients with AF and the development of endovascular treatment (EVT) for patients with large vessel occlusion (LVO)‐AIS. While ischemic stroke occurs only in 2% of patients on anticoagulant treatment per year,^10^ 38% of patients with AIS and AF receive prior OACs.^11^ The number of patients who receive DOACs before EVT for LVO‐AIS has dramatically increased.^12^ Patients who develop AIS with LVO despite prior use of OACs for stroke prevention may have the opportunity to receive EVT in several facilities.

Consequently, it is important for modern clinicians to determine the relationships between the CHA_2_DS_2_‐VASc score, prior use of OACs, and the outcomes after EVT. In this study, we aimed to assess the additional impact of OACs on EVT, mainly focusing on pre‐stroke CHA_2_DS_2_‐VASc scores.

## Methods

### Study design and data source

Patients were selected from the third cohort (from January 2018 to March 2020) of the Close The Gap‐Stroke program, the largest nationwide quality improvement program in Japan in the J‐ASPECT study (a nationwide survey of Acute Stroke Care Capacity for Proper Designation of Comprehensive Stroke Center in Japan).^13^ The rationale and full study design have been published elsewhere.^13^, ^14^ The data supporting the present findings are available from the corresponding author upon reasonable request. The present study conformed to the STROBE (The Strengthening the Reporting of Observational Studies in Epidemiology) Reporting Guidelines.

This study identified consecutive patients with AIS in the third cohort of the Close The Gap‐Stroke program who were aged ≥18 years and received EVT from the diagnosis procedure combination claim database of 416 primary stroke centers. Patients who met the following criteria were included: (i) duration from the last time known to be well ≤6 h and (ii) history of AF or newly diagnosed AF after stroke onset. Patients whose last time known to be well, OAC, or CHA_2_DS_2_‐VASc score information was not available were excluded.

All the patients were categorized into three mutually exclusive groups based on the preceding OAC treatment: warfarin, DOAC, and non‐OAC groups. The following baseline data were collected based on the electronic medical records and data from the health insurance claims database: age, sex, stroke risk factor (hypertension [HT], diabetes mellitus, dyslipidemia, AF before stroke, and smoking), other factors associated with CHA_2_DS_2_‐VASc (congestive heart failure or left ventricular dysfunction, age ≥75 years, stroke or transient ischemic attack, myocardial infarction, or peripheral vascular disease, age 65–74 years), international normalized ratio, serum creatinine, body mass index, and pre modified Rankin Scale. The following AIS data were collected: time from onset to door of ≤3.5 h, time from door to puncture, baseline National Institutes of Health Stroke Scale score, Alberta Stroke Program Early CT Score (ASPECTS), occlusion site (LVO: internal carotid artery, M1, A1, vertebral artery, basilar artery, or P1, or primary medium vessel occlusion), anticoagulant antagonist use, and intravenous tissue‐type plasminogen activator use.

Patients were also classified into two categories based on the pre‐stroke CHA_2_DS_2_‐VASc score in the primary analysis. The patients with CHA_2_DS_2_‐VASc scores of ≥2 for males and ≥3 for females were allocated to the high‐risk group according to the 2023 ACC/AHA/ACCP/HRS and 2020 ESC guideline.^2^, ^4^ Those with scores of ≤1 for males or ≤2 for females were allocated to the integrated intermediate‐ to low‐risk group.

### Outcome measures

The safety outcomes were symptomatic intracerebral hemorrhage (sICH) within 36 h after EVT and in‐hospital mortality, while the efficacy outcomes were functional independence at 90 days, successful reperfusion, and complete reperfusion. sICH was defined according to the European Cooperative Acute Stroke Study II (ECASS‐II) as clinical worsening of at least 4 points on the National Institutes of Health Stroke Scale, attributed to parenchymal hematoma, subarachnoid, or intraventricular hemorrhage.^15^ Functional independence was defined as modified Rankin Scale scores of 0–2 at 90 days. Successful and complete reperfusion were defined as modified thrombolysis in cerebral infarction (mTICI) grades of ≥2b and mTICI grades 3, respectively.

### Statistical analysis

The baseline characteristics of the three groups based on preceding OAC treatment were summarized. Continuous variables were presented as median (inter quartile range [IQR]), and categorical variables are presented as frequency (%).

The following analyses were conducted using a multiple mixed‐effects logistic regression model, with the hospital unit ID codes as a random effect. The rationale of the pre‐specified model was to adjust for known outcome predictors. These included age, female sex, body mass index, HT, National Institutes of Health Stroke Scale (NIHSS) score, ASPECTS score, tissue‐type plasminogen activators for sICH, in‐hospital mortality, and functional independence at 90 days. For successful and complete reperfusion, serum creatinine level, and LVO or medium vessel occlusion were included instead of body mass index and HT as adjustment variables.

As a preliminary analysis, the relationships between the outcomes and CHA_2_DS_2_‐VASc score as continuous variables were analyzed. In this analysis, anticoagulants treatment groups (warfarin, DOACs, or non‐OACs) was added as an adjustment variable. For the primary analysis, the relationships between prior OAC use and the outcomes were analyzed separately for the two risk groups (high and intermediate to low) based on the CHA_2_DS_2_‐VASc score. A subgroup analysis was conducted to assess how the eight factors constituting the CHA_2_DS_2_‐VASc modified the impact of prior OAC use on the outcomes. The eight subgroup analyses were additionally adjusted for the total CHA_2_DS_2_‐VASc score, while the adjustment variables constituting CHA_2_DS_2_‐VASc, such as age, female sex, and HT were excluded from the baseline model.

Statistical significance was set at *p* values of <0.05. All statistical analyses were performed using the R software, version 4.1.1 (R Foundation for Statistical Computing, Vienna, Austria).

## Results

### Study population and baseline characteristics

**Figure 1:**
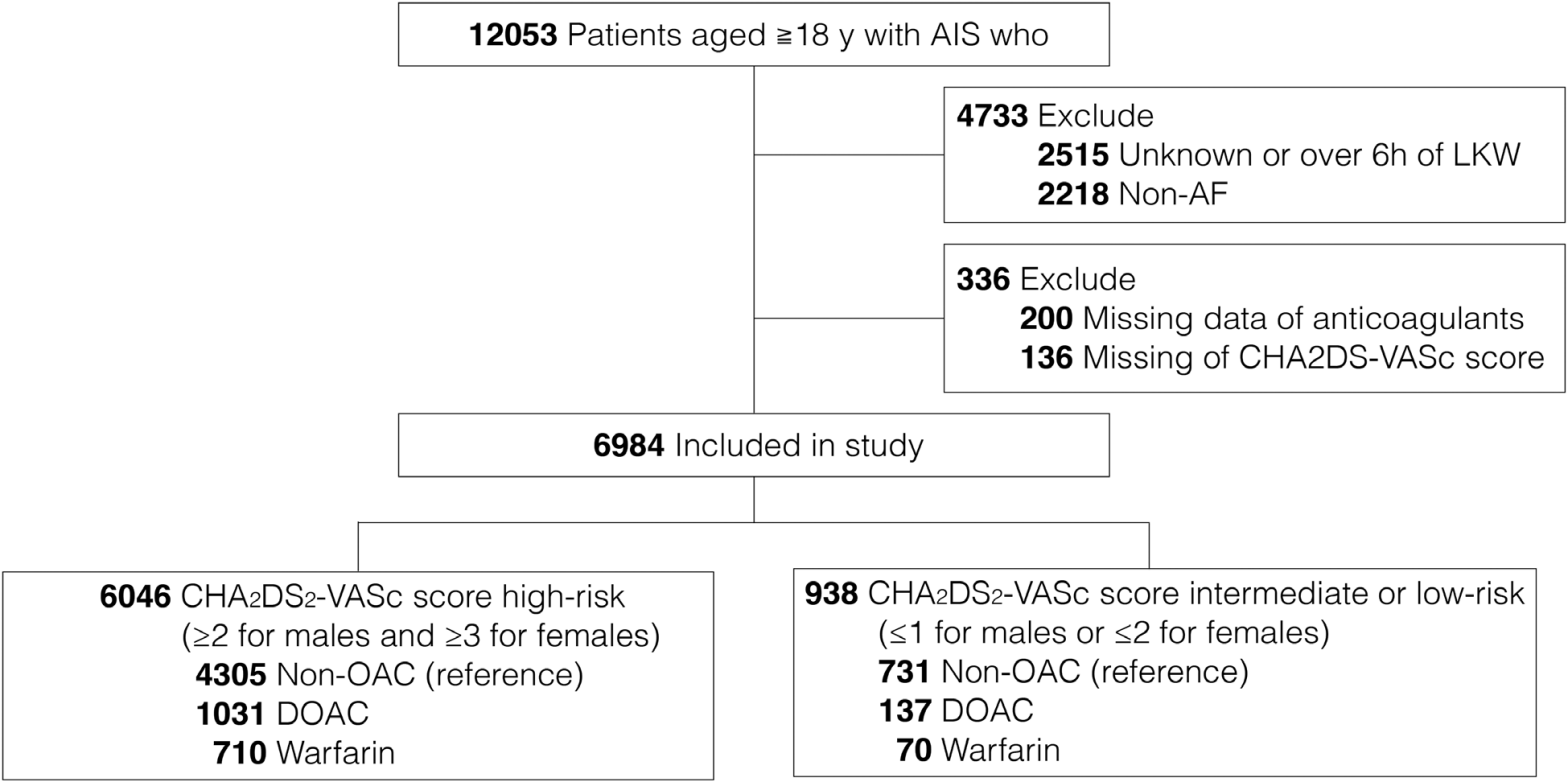
Patient selection flowchart. AF, atrial fibrillation; AIS, acute ischemic stroke; DOAC, direct oral anticoagulants; LKW, duration from the last time known to be well.

A total of 12053 patients who underwent EVT at 353 centers were identified via the Close The Gap‐Stroke program. Among these, 5069 patients were excluded due to unknown data on the last time known to be well or durations from the last time known to be well >6 h (*n* = 2315), non‐AF (*n* = 2218), and missing data on prior OAC use (*n* = 200) and CHA_2_DS_2_‐VASc scores (*n* = 136). Finally, 6984 patients met the inclusion criteria. Their median age (IQR) was 80 (73–86) years, and 48.5% were female. The patient selection flowchart is shown in Figure 1.

Among the 6984 patients, 780 (11.2%) and 1168 (16.7%) patients received warfarin and a DOAC, respectively, and 5036 (72.1%) did not use OAC before stroke onset. The baseline characteristics of each OAC group are summarized in Table 1. The warfarin and DOAC users had a higher prevalence of previous HT, AF, congestive heart failure, and vascular disease than those who did not use OAC. Warfarin users showed especially higher prevalence of DM, dyslipidemia, congestive heart failure, and renal disease than the other two groups. They also more frequently underwent CT for ASPECTS evaluation and detection of the occlusion site of the ICA and used anticoagulant antagonists. Further, they had higher international ratios and serum creatinine concentrations and less frequently used intravenous tissue‐type plasminogen activators. The median (IQR) CHA_2_DS_2_‐VASc score was 3 (2–4) for non‐OAC and DOAC user and 3 (3, 4) for warfarin user.

**Table 1:** Baseline characteristics stratified by prior OAC use.

Characteristics	Non‐OAC (*n* = 5036)	Warfarin (*n* = 780)	DOAC (*n* = 1168)
Baseline characteristics			
Age, median (IQR), years	80.0 [72.0–86.0]	80.5 [74.0–86.0]	80.0 [74.0–85.0]
Female, no. (%)	2452 (48.7)	391 (50.1)	544 (46.6)
Hypertension, no. (%)	2675 (53.1)	443 (56.8)	664 (56.8)
Diabetes mellitus, no. (%)	878 (17.4)	169 (21.7)	201 (17.2)
Dyslipidemia, no. (%)	991 (19.7)	183 (23.5)	240 (20.5)
Atrial fibrillation (before stroke), no. (%)	3469 (68.9)	633 (81.2)	999 (85.5)
Current/past smoker, no. (%)	5455 (11.9)	62 (8.5)	104 (9.8)
CHA_2_DS_2_‐VASc score, median (IQR)	3 [2–4]	3 [3–4]	3 [2–4]
CHF or left ventricular dysfunction, no. (%)	627 (12.5)	182 (23.3)	189 (16.2)
Age ≥75 years, no. (%)	3470 (68.9)	565 (72.4)	853 (73.0)
Stroke or transient ischemic attack, no. (%)	375 (7.4)	50 (6.4)	77 (6.6)
MI or peripheral vascular disease, no. (%)	236 (4.7)	52 (6.7)	62 (5.3)
Age 65–74 years, no. (%)	1082 (21.5)	162 (20.8)	250 (21.4)
International normalized ratio, median (IQR)	1.03 [0.97–1.10]	1.37 [1.17–1.67]	1.10 [1.02–1.25]
Serum creatinine, median (IQR), mg/dL	0.84 [0.69–1.05]	0.94 [0.76–1.25]	0.85 [0.70–1.04]
Body mass index, median (IQR), kg/m^2^	22.4 [20.0–24.7]	21.7 [19.1–24.6]	22.6 [20.2–24.7]
Pre modified Rankin Scale, median (IQR)	0 [0–1]	1 [0–2]	0 [0–2]
Stroke characteristics			
Onset to door ≤3.5 h, no. (%)	4439 (90.6)	694 (91.0)	1045 (91.4)
Door to puncture, median (IQR), min	75 [52–106]	77 [54–110]	78 [54–110]
Baseline NIHSS, median (IQR)	18 [13–24]	20 [14–25]	19 [13–24]
ASPECTS, median (IQR)	8 [6–10]	8 [6–10]	8 [6–10]
Occlusion site, no. (%)			
Large vessel occlusion	3960 (79.2)	631 (81.7)	913 (79.3)
Internal carotid artery	1262 (25.2)	260 (33.7)	311 (27.0)
M1	2609 (52.2)	379 (49.1)	575 (49.9)
A1	159 (3.2)	28 (3.6)	39 (3.4)
Vertebral artery	54 (1.1)	11 (1.4)	15 (1.3)
Basilar artery	309 (6.2)	51 (6.6)	94 (8.2)
P1	96 (1.9)	20 (2.6)	27 (2.3)
Primary medium vessel occlusion	1040 (20.8)	141 (18.3)	239 (20.7)
Anticoagulant antagonist use, no. (%)	116 (2.3)	93 (11.9)	28 (2.4)
Intravenous tPA use, no. (%)	2853 (56.7)	302 (38.7)	526 (45.0)

ASPECTS, Alberta Stroke Program Early CT Score; CHF, congestive heart failure; DOAC, direct oral anticoagulants; IQR, interquartile range; MI, myocardial infarction; NIHSS, National Institutes of Health Stroke Scale; OAC, oral anticoagulants; tPA, tissue type plasminogen activator.

### CHA_2_DS_2_ ‐VASc score and outcome measures

**Figure 2:**
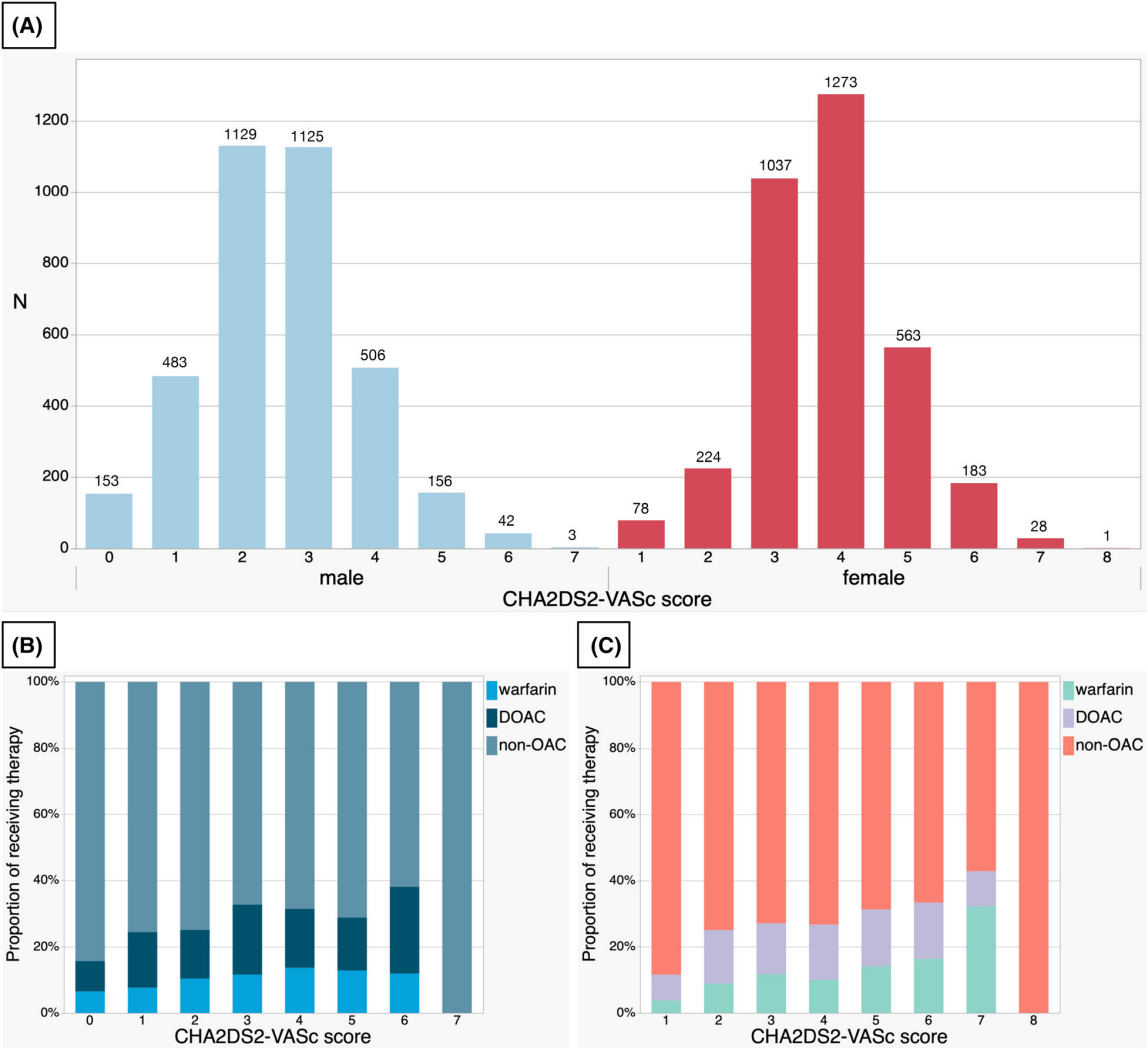
The distribution of CHA_2_DS_2_‐VASc score and prevalence of prior DOAC use by gender. (A) The distribution of patients with AF who underwent EVT based on the CHA_2_DS_2_‐VASc score by sex is shown. (B and C) The prevalence of OAC treatment and the proportions of warfarin and DOACs are shown by gender. DOAC, direct oral anticoagulants; OAC, oral anticoagulant.

CHA_2_DS_2_‐VASc scores representing a high‐risk and intermediate‐ or low‐risk were determined for 6046 (86.6%) and 938 (13.4%) of the patients, respectively. The prevalence of OAC use tended to increase with increasing CHA_2_DS_2_‐VASc score (Fig. 2). The incidence of sICH and in‐hospital mortality, functional independence at 90 days, successful reperfusion, and complete reperfusion rates were 660/6982 (9.5%), 764/6984 (10.9%), 1910/4914 (38.9%), 5839/6983 (83.6%), and 3327/6984 (47.6%), respectively. Higher CHA_2_DS_2_‐VASc scores, as continuous data, were associated with increased risk of sICH (adjusted odds ratio [aOR] [95% CI], 1.21 [1.07–1.37], *p* = 0.003), in‐hospital mortality (aOR [95% CI], 1.19 [1.06–1.33], *p* = 0.003), and decreased functional independence at 90 days (aOR [95% CI], 0.87 [0.79–0.96], *p* = 0.007). There were no significant associations between the CHA_2_DS_2_‐VASc score and successful (aOR [95% CI], 1.02 [0.94–1.10], *p* = 0.67) and complete (aOR [95% CI], 1.00 [0.95–1.06], *p* = 0.92) reperfusions (Table 2).

**Table 2:** Odds ratios of the outcomes by CHA_2_DS_2_‐VASc as continuous variable.

	Outcome measure				
	sICH^a^	In‐hospital mortality^a^	Functional independence at 90 days^a^	Successful reperfusion (mTICI ≥2b)^b^	Complete reperfusion (mTICI = 3)^b^
No./total no. (%)	660/6982 (9.5)	764/6984 (10.9)	1910/4914 (38.9)	5839/6983 (83.6)	3327/6984 (47.6)
CHA_2_DS_2_‐VASc score (as continuous variable)					
Unadjusted OR (95% CI)	1.12 (1.06–1.19)	1.21 (1.14–1.28)	0.67 (0.64–0.70)	0.86 (0.82–0.90)	0.96 (0.92–0.99)
*p* value	<0.001	<0.001	<0.001	<0.001	0.01
Adjusted OR (95% CI)	1.21 (1.07–1.37)	1.19 (1.06–1.33)	0.87 (0.79–0.96)	1.02 (0.94–1.10)	1.00 (0.95–1.06)
*p* value	0.003	0.003	0.007	0.67	0.92

ASPECTS, Alberta Stroke Program Early CT Score; CI, confidence interval; mTICI, modified thrombolysis in cerebral infarction; OR, odds ratio; sICH, symptomatic intracerebral hemorrhage.

^a^ Adjusted with age; female sex; body mass index; hypertension; National Institutes of Health Stroke Scale; ASPECTS; tissue‐type plasminogen activators; anticoagulant treatment group.

^b^ Adjusted with age; female sex; serum creatinine; National Institutes of Health Stroke Scale; ASPECTS; large vessel occlusion; tissue‐type plasminogen activators; anticoagulant treatment group.

### Prior OAC use, CHA_2_DS_2_ ‐VASc score, and outcome measures

For the high‐risk patients, compared with the non‐OAC user, warfarin use was associated with increased prevalence of sICH (90 of 710 [12.7%] vs. 412 of 4304 [9.6%], unadjusted OR [95% CI], 1.37 [1.08–1.75], aOR [95% CI], 1.50 [1.10–2.05], *p* = 0.01) and decreased functional independence at 90 days (128 of 501 [25.5%] vs. 1116 of 3026 [36.9%], unadjusted OR [95% CI], 0.59 [0.47–0.73], aOR [95% CI], 0.63 [0.47–0.83], *p* < 0.001) (Table 3). DOAC use, compared with non‐OAC use, was associated with increased successful reperfusion (870 of 1031 [84.4%] vs. 3560 of 4305 [82.7%], unadjusted OR [95% CI], 1.13 [0.94–1.36], aOR [95% CI], 1.27 [1.00–1.61], *p* = 0.047) and complete reperfusion (530 of 1031 [51.4%] vs. 2002 of 4305 [46.5%] unadjusted OR [95% CI], 1.22 [1.06–1.40], aOR [95% CI], 1.30 [1.10–1.53], *p* = 0.002) rates in the adjusted model. However, no association was observed between prior use of DOACs and other outcomes (sICH, in‐hospital mortality, and functional independence at 90 days).

**Table 3:** Odds ratios of the outcomes stratified by prior OAC use for patients with high‐risk CHA_2_DS_2_‐VASc scores.

Outcome measure	High‐risk CHA_2_DS_2_‐VASc score (≥2 for males, ≥3 for females)				
	Non‐OAC *n* = 4305	Warfarin *n* = 710	*p* value	DOAC *n* = 1031	*p* value
sICH					
No./total (%)	412/4304 (9.6)	90/710 (12.7)		94/1031 (9.1)	
Unadjusted OR (95% CI)	Reference	1.37 (1.08–1.75)	0.01	0.94 (0.75–1.20)	0.65
Adjusted OR (95% CI)^a^	Reference	1.50 (1.10–2.05)	0.01	1.13 (0.83–1.52)	0.44
In‐hospital mortality					
No./total (%)	517/4305 (12.0)	98/710 (13.8)		96/1031 (9.3)	
Unadjusted OR (95% CI)	Reference	1.17 (0.93–1.48)	0.18	0.75 (0.60–0.95)	0.01
Adjusted OR (95% CI)^a^	Reference	1.20 (0.89–1.61)	0.23	0.85 (0.64–1.14)	0.29
Functional independence at 90 days					
No./total (%)	1116/3026 (36.9)	128/501 (25.5)		257/715 (35.9)	
Unadjusted OR (95% CI)	Reference	0.59 (0.47–0.73)	<0.001	0.96 (0.81–1.14)	0.61
Adjusted OR (95% CI)^a^	Reference	0.63 (0.47–0.83)	<0.001	0.88 (0.69–1.11)	0.27
Successful reperfusion (mTICI ≥2b)					
No./total (%)	3560/4305 (82.7)	574/709 (81.0)		870/1031 (84.4)	
Unadjusted OR (95% CI)	Reference	0.89 (0.73–1.09)	0.26	1.13 (0.94–1.36)	0.20
Adjusted OR (95% CI)^b^	Reference	0.91 (0.71–1.17)	0.47	1.27 (1.00–1.61)	0.047
Complete reperfusion (mTICI = 3)					
No./total (%)	2002/4305 (46.5)	323/71 (45.5)		530/1031 (51.4)	
Unadjusted OR (95% CI)	Reference	0.96 (0.82–1.13)	0.62	1.22 (1.06–1.40)	0.005
Adjusted OR (95% CI)^b^	Reference	0.97 (0.80–1.17)	0.74	1.30 (1.10–1.53)	0.002

ASPECTS, Alberta Stroke Program Early CT Score; CI, confidence interval; DOAC, direct oral anticoagulants; mTICI, modified thrombolysis in cerebral infarction; OAC, oral anticoagulants; OR, odds ratio; sICH, symptomatic intracerebral hemorrhage.

^a^ Adjusted with age; female sex; body mass index; hypertension; National Institutes of Health Stroke Scale; ASPECTS; tissue‐type plasminogen activators.

^b^ Adjusted with age, female sex; serum creatinine; National Institutes of Health Stroke Scale; ASPECTS; large vessel occlusion; tissue‐type plasminogen activators.

For the intermediate‐ or low‐risk patients, compared with the non‐OAC user, there was no significant associated between OAC use and outcomes (Table 4).

**Table 4:** Odds ratios of the outcomes stratified by prior OAC use for patients with intermediate‐ to low‐risk CHA_2_DS_2_‐VASc scores.

Outcome measure	Intermediate‐ to low‐risk CHA_2_DS_2_‐VASc score (≤1 for males, ≤2 for females)				
	Non‐OAC *n* = 731	Warfarin *n* = 70	*p* value	DOAC *n* = 137	*p* value
sICH					
No./total (%)	56/730 (7.7)	3/70 (4.3)		5/132 (3.6)	
Unadjusted OR (95% CI)	Reference	0.54 (0.16–1.77)	0.31	0.46 (0.18–1.16)	0.10
Adjusted OR (95% CI)^a^	Reference	0.51 (0.12–2.22)	0.37	0.25 (00.06–1.07)	0.06
In‐hospital mortality					
No./total (%)	44/731 (6.0)	4/70 (5.7)		5/137 (3.6)	
Unadjusted OR (95% CI)	Reference	0.95 (0.33–2.72)	0.92	0.59 (0.23–1.52)	0.28
Adjusted OR (95% CI)^a^	Reference	0.34 (0.04–2.61)	0.30	0.66 (0.18–2.38)	0.52
Functional independence at 90 days					
No./total (%)	331/537 (61.6)	33/49 (67.3)		45/86 (52.3)	
Unadjusted OR (95% CI)	Reference	1.28 (0.69–2.39)	0.43	0.68 (0.43–1.08)	0.10
Adjusted OR (95% CI)^a^	Reference	1.26 (0.53–3.01)	0.60	0.56 (0.29–10.08)	0.08
Successful reperfusion (mTICI ≥2b)					
No./total (%)	657/731 (89.9)	63/70 (90.0)		115/137 (83.9)	
Unadjusted OR (95% CI)	Reference	1.01 (0.45–2.29)	0.97	0.59 (0.35–0.99)	0.04
Adjusted OR (95% CI)^b^	Reference	1.27 (0.39–4.09)	0.69	0.62 (0.28–1.36)	0.23
Complete reperfusion (mTICI = 3)					
No./total (%)	374/731 (51.2)	36/70 (51.4)		62/137 (45.3)	
Unadjusted OR (95% CI)	Reference	1.01 (0.62–1.65)	0.97	0.79 (0.55–1.14)	0.21
Adjusted OR (95% CI)^b^	Reference	1.04 (0.55–1.95)	0.91	0.70 (0.42–1.15)	0.16

ASPECTS, Alberta Stroke Program Early CT Score; CI, confidence interval; DOAC, direct oral anticoagulants; mTICI, modified thrombolysis in cerebral infarction; OAC, oral anticoagulants; OR, odds ratio; sICH, symptomatic intracerebral hemorrhage.

^a^ Adjusted with age; female sex; body mass index; hypertension; National Institutes of Health Stroke Scale; ASPECTS; tissue‐type plasminogen activators.

^b^ Adjusted with age, female sex; serum creatinine; National Institutes of Health Stroke Scale; ASPECTS; large vessel occlusion; tissue‐type plasminogen activators.

### Subgroup analyses

**Figure 3:**
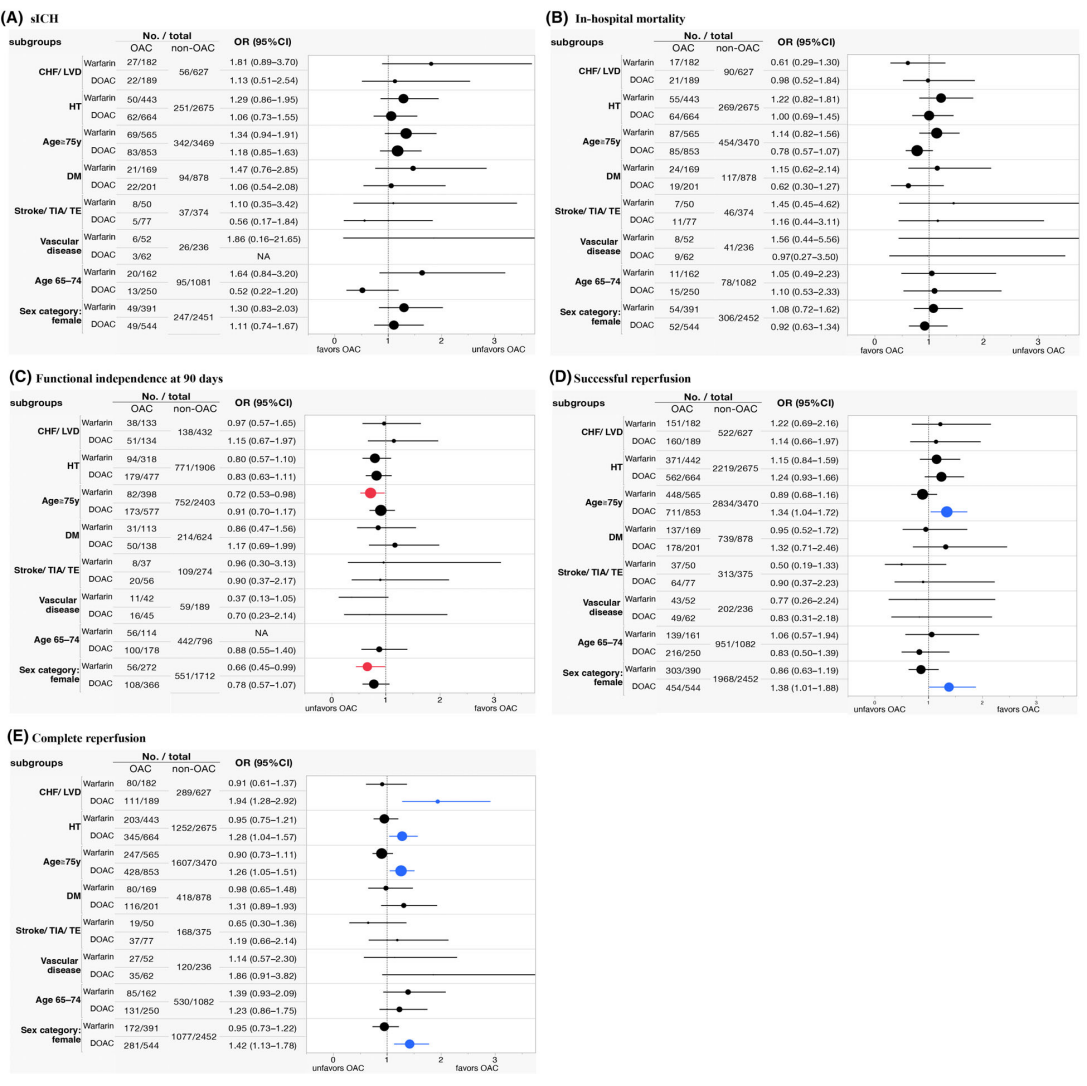
Subgroup analyses of the eight factors constituting the CHA_2_DS_2_‐VASc. The marker size represents the total number of participants in each subgroup. The red marker color represents a significant relationship between prior OAC use and unfavorable outcomes while the blue color represents the relationship with favorable outcomes. (A) Analysis for sICH. (B) Analysis for in‐hospital mortality. (C) Analysis for functional independence at 90 days. (D) Analysis for successful reperfusion. (E) Analysis for complete reperfusion. CHF, congestive heart failure; CI, confidence interval; DM, diabetes mellitus; DOAC, direct oral anticoagulants; HT, hypertension; LVD, left ventricular dysfunction; OAC, oral anticoagulants; OR, odds ratio; sICH, symptomatic intracerebral hemorrhage; TE, thromboembolism; TIA, transient ischemic attack.

The subgroup analyses based on the factors constituting CHA_2_DS_2_‐VASc are shown in Figure 3. Warfarin use was associated with decreased functional independence at 90 days among the patients with age ≥75 years (aOR [95% CI], 0.72 [0.53–0.98]) and sex category: female (aOR [95% CI], 0.66 [0.45–0.99]) using non‐OAC use as a reference. DOAC use was associated with an increase in the rates of successful reperfusion among the patients with age ≥75 years (aOR [95% CI], 1.34 [1.04–1.72]) and sex category: female (aOR [95% CI], 1.38 [1.01–1.8]) and complete reperfusion among the patients with congestive heart failure or left ventricular dysfunction (aOR [95% CI], 1.94 [1.28–2.92]), HT (aOR [95% CI], 1.28 [1.04–1.57]), age ≥75 years (aOR [95% CI], 1.26 [1.05–1.51]), and sex category: female (aOR [95% CI], 1.42 [1.13–1.78]) using non‐OAC user as a reference.

## Discussion

The major findings of this study were that, although higher CHA_2_DS_2_‐VASc scores were associated with worse clinical outcomes after EVT, prior DOAC use improved the successful and complete reperfusion rates among the patients with high‐risk CHA_2_DS_2_‐VASc scores. Meanwhile, prior warfarin use increased the risk of sICH and decreased of functional independence at 90 days. For patients with intermediate‐to‐low‐risk CHA_2_DS_2_‐VASc scores, prior OAC use did not have any impact on outcomes. Additionally, regardless of total CHA_2_DS_2_‐VASc scores, patients with congestive heart failure or left ventricular dysfunction, HT, age >75 years, or sex category: female benefited similarly from prior DOAC use. Patients with age >75 years, age 65–74, or sex category: female may have decreased functional independence with prior warfarin use. These findings may provide clinicians with supplemental evidence to guide the introduction of DOACs based on the CHA_2_DS_2_‐VASc score for high‐ and selected intermediate‐risk patients.

Studies have revealed that prior warfarin use increases the risk of sICH after EVT^16^, ^17^, ^18^, ^19^ or that neither prior warfarin nor DOAC use affects outcomes.^20^, ^21^, ^22^, ^23^, ^24^, ^25^ Our study revealed an unprecedented large number of patients from a nationwide cohort in East Asia; the impact of OACs on the outcomes after EVT varies according to the CHA_2_DS_2_‐VASc score. Additionally, as preliminary analyses revealed, the CHA_2_DS_2_‐VASc score affected the outcomes after EVT, regardless of the prior OAC use. We need to establish that a higher CHA_2_DS_2_‐VASc score is a risk factor for sICH, in‐hospital mortality, and poor functional outcomes after EVT in patients with AIS.

Two mechanisms may explain the increased successful and complete reperfusion rate after prior DOAC use. The first is the change in the volume of the thrombus. DOACs prolongs clot formation time, which may provide a relatively longer time for a small thrombus to escape from the atrium, preventing large thrombi formation.^26^ Additionally, less secondary thrombi around occluded site seems to be formed under the anticoagulated status. It is well‐known that higher clot burden score, meaning smaller volume thrombus, is associated with successful reperfusion.^27^ The effect of DOACs on thrombus components is the second hypothesis. Wang et al. showed that DOACs caused significant changes in the thrombus composition, with an increase in the fibrin and platelet content, resulting in increased trend of successful reperfusion.^28^ Fibrin‐rich thrombi led higher fracture toughness than red blood cell‐rich thrombi.^29^ These specific thrombi characteristic in DOAC users may explain decrease of embolization to new territory, resulting in increased complete reperfusion in our study. Although fibrin‐rich thrombi have been reported as a risk factor of failure of reperfusion,^30^ the interpretation might be influenced by the confounder such as various underlying stroke etiology. Our hypothesis may be true only among the patients with AF. The combination of relatively smaller size and higher fracture toughness in thrombi caused by DOAC may improve EVT outcomes. The lack of effects of prior DOAC use on clinical outcomes at 90 days in these high‐risk patients, despite increased reperfusion, deserves attention. The “no reflow” phenomenon is considered as an important hypothesis to explain this discrepancy. No reflow phenomenon means the resultant distal hypoperfusion due to dysfunction of the microvasculature despite macrovascular recanalization.^31^ The underlying mechanisms are multifaceted, encompassing the formation of microemboli, microvascular compression, and contraction.^32^ Discrepancy between successful reperfusion and functional failure after thrombolysis and thrombectomy for LVO‐AIS, is often referred to as the no reflow phenomenon. In patients with acute coronary syndrome, a higher CHA_2_DS_2_‐VASc score was associated with the no reflow phenomenon after the percutaneous intervention.^33^ The discrepancy in our study may suggest the increased prevalence of underlying “no reflow” phenomenon in patient with higher CHA_2_DS_2_‐VASc score. Contrastingly, prior use of DOAC in patients with intermediate‐ or low‐risk CHA_2_DS_2_‐VASc scores did not show additional efficacy in this study.

Our study also showed that prior warfarin treatment in patients with high‐risk CHA_2_DS_2_‐VASc scores increased the risk of sICH and decreased functional independence after EVT. A previous study showed that a higher CHA_2_DS_2_‐VASc score was associated with a higher prevalence of cerebral micro bleeds and higher HASBLED scores.^34^ The combination of cerebral microbleeds and warfarin use increases the risk of intracerebral hemorrhage.^35^ Additionally, patients who introduced not DOACs but warfarin seem likely to have more complex back grounds. In fact, warfarin users were more likely to have DM, dyslipidemia, CHF, renal disease, anticoagulant agonist use, and not receive tPA in our study. Although tPA use, and DM and CHF as components of CHA_2_DS_2_‐VASc were included in the adjusted model, we may have to interpret our result with the possibility that the pure impact of warfarin might be overestimated. These patients may have specific indications for the introduction of warfarin, such as dialysis or mechanical valves, but it is important to recognize that the combination of a CHA_2_DS_2_‐VASc high score and prior use of warfarin increases sICH and results in decreased functional independence after EVT.

The number of patients receiving OAC has been increasing in the super‐aging societies, such as Japan. Our nationwide study showed that 27.9% of patients with AF who received EVT were OAC users. This highlights the need to better understand the effect of prior OAC use based on CHA_2_DS_2_‐VASc score on prevention, disease severity, and treatment outcomes in patients with AIS.

## Limitations and Strengths

This study has several limitations. First, the Japanese population with AF has a relatively lower risk of ischemic stroke than other populations.^36^ The present results may not be generalizable to other ethnic groups. Second, AF known before stroke and that detected after stroke were not distinguished in this study. However, Lyer F et al. revealed that they are clinically relevant.^37^ Third, it is uncertain whether the etiology of AIS was only cardiogenic embolism for patients with AF, especially those with low‐risk CHA_2_DS_2_‐VASc scores. Forth, this was a retrospective study that assessed the validity and additional impact of OACs. A prospective study is needed to clarify the net clinical benefit for ischemic stroke, as well as all major adverse events. Fifth, there may have been selection bias in the hospitals that participated in this study.^13^

This study had several strengths. First, it was based on the largest nationwide real‐world dataset collected from stroke centers in Japan. The Close The Gap‐Stroke Program is the largest nationwide quality improvement initiative in Japan. Second, an association between stroke quality indicators and clinical outcomes in patients with AIS who participated in this program was recently reported. Third, the large number of participants in the most recent study period enables the analysis of the additional impact of OACs on EVT using modern devices and based on stratification by the CHA_2_DS_2_‐VASc score.

## Conclusion

In this largest nationwide study, prior DOAC use by patients with high‐risk CHA_2_DS_2_‐VASc scores improved angiographical outcomes of EVT without the risk of increased sICH. These findings may provide supplemental evidence to guide clinicians in deciding to introduce preventive DOAC for patients with AF in this EVT era. However, prior DOAC use did not improve the functional outcomes for patients who received EVT, suggesting a need for a pivotal strategy for improving the prognosis of LVO‐AIS.

## Author Contributions

Y.I., R.N., S.O., S.W., and K.I. contributed to the conception and design of the study; all authors contributed acquisition and analysis of data; Y.I., R.N., S.O., and K.I. contributed to drafting a significant portion of the manuscript or figures.

## Funding Information

This work was supported by the Practical Research Project for lifestyle‐related diseases, including cardiovascular diseases and diabetes mellitus, managed by the Japan Agency for Medical Research and Development (JP19ek0210088, JP20ek0210129, JP20ek0210147, JP21ek0210147, and JP22ek0210147); Grants‐in‐Aid from the Japanese Ministry of Health, Labour and Welfare (H28‐Shinkin‐Ippan‐011, 19AC1003, 21FA1010, 22FA1015, 23FA1014, 24FA1015, and 24FA1016); Grants‐in‐Aid for Scientific Research (KAKENHI) (25293314, 18H02914, 22H03191, and 23K24450; principal investigator: Koji Iihara) from the Japan Society for the Promotion of Science; and Intramural Research Fund (20‐4‐10) for Cardiovascular Diseases of National Cerebral and Cardiovascular Center.

## Conflict of Interest

Dr. Imamura: speakers' bureau/honoraria from Medtronic Japan Co. (which own patent rights to EVT device), Daiichi Sankyo Co. (which own patent rights to DOAC edoxaban), Johnson & Johnson Co. (which own patent rights to EVT device), Stryker Japan Co. (which own patent rights to EVT device), Terumo Co. (which own patent rights to EVT device), and Asahi Intecc Co. (which own patent rights to EVT device). Dr. Ihara: lecturer's fees from Daiichi Sankyo Co. and Eisai (which own patent rights to warfarin), and Bristol‐Myers Squibb (which own patent rights to DOAC apixaban). Dr. Kamiyama: lecturer's fees from Daiichi Sankyo Co. Dr. Ohta reported lecturer's fees from Medtronic, Daiichi‐Sankyo, Johnson & Johnson, Terumo, Stryker Japan, Tokai Medical (which own patent rights to EVT device), Otsuka (which own patent rights to warfarin), Takeda (which own patent rights to warfarin), Eisai (which own patent rights to anticoagulant antagonist), Kaneka (which own patent rights to EVT device), Bristol‐Myers Squibb, AstraZeneca (which own patent rights to anticoagulant antagonist), Japan Lifeline (which own patent rights to EVT device), Nipro (which own patent rights to EVT device), and Century Medical (which own patent rights to EVT device), as well as consulting fees for Johnson & Johnson and Tokai Medical. Dr. Endo: lecturer's fees from Daiichi Sankyo. Dr Matsumaru: lecturer fees from Medtronic, Stryker, Terumo, Kaneka, and Daiichi Sakyo. Dr Sakai: a research grant from Japan Lifeline, Kaneka, Medtronic, Terumo, and TG Medical (which own patent rights to EVT device); lecturer's fees from Asahi‐Intec, Kaneka, Medtronic, Stryker, and Terumo; membership on the advisory boards for Johnson & Johnson, Medtronic, and Terumo. Dr. Kitazono: lecturer's fees and a research grant from Daiichi Sankyo. The remaining authors declare no conflicts of interest.

## Supporting information


Data S1.

## Data Availability

The data that support the findings of this study are available from the corresponding author upon reasonable request.
