# Supplementary material for: CHA2DS2 ‐VASc score and prior oral anticoagulant use on endovascular treatment for acute ischemic stroke
Source: Ann Clin Transl Neurol. 2024 Oct 9;11(12):3103–14. doi: 10.1002/acn3.52217 (PMC11651198; doi:10.1002/acn3.52217)
Supplement: Supplementary file 1 — Data S1. [file ACN3-11-3103-s001.docx]

**The name of hospitals and Collaborators with 3^rd^ cohort of Close The Gap-Stroke, J-ASPECT Study.**

| Name of institutions | Name of collaborators |
| --- | --- |
| Ageo Central General Hospital | Takashi Shimizu |
| Ainomiyako　Neurosurgical　Hospital | Isao Sasaki |
| Akita Cerebrospinal and Cardiovascular Center | Junta Moroi |
| Akita City Hospital | Satoshi Okawa |
| Akita University Hospital | Hiroaki Shimizu |
| Ako City Hospital | Minoru Asahi |
| Almeida Memorial Hospital | Makoto Goda |
| Amagasaki Chuou Hospital | Tsuyoshi Mtsumoto |
| Anjo Kosei Hospital | Takahisa Kano |
| Aomorishintoshi Hospital | Yoichi Katayama |
| Arao Municipal Hospital | Takamasa Mizuno |
| Asahi General Hospital | Shigeru Oya |
| Asahikawa Medical University Hospital | Manabu Kinoshita |
| Asahikawa Red Cross Hospital | Takizawa Katsumi |
| Asakuraisikai Hospital | Takao Ooasa |
| Azuma Neurosurgical Hospital | Taku Sato |
| Baba Memorial Hospital | Yukihide Kanemoto |
| Bellland General Hospital | Ryuunosuke Uranishi |
| Brain Attack Center Ota Memorial Hospital | Yuka Terasawa |
| Center Hospital of The National Center for Global Health and Medicine | Tetsuo Hara |
| Chiba Central Medical Center | Motoki Sato |
| Chiba Emergency Medical Center | Toshihiro Yamauchi |
| Chiba Medical Center | Ken Kado |
| Chiba Neurosurgical Clinic | Kenji Wakui |
| Chibaken Saiseikai Narashino Hospital | Atsushi Fujikawa |
| Chibanishi General Hospital | Junichiro Kumai |
| Chibune General Hospital | Okada Takashi |
| Chikamori Hospital | Satoru Hayashi |
| Chubu Tokushyukai Hospital | Tatuya Sihngaki |
| Chuno Hospital | Shinji Noda |
| Chutoen General Medical Center | Takeshi Torigai |
| Daido Hospital | Takashi Tsujiuchi |
| Daiyukai General Hospital | Takayuki Kato |
| Department of Neurosurgery Graduate School of Medical Science Kanazawa University | Mitsutoshi Nakada |
| Eastern Chiba Medical Center | Toshio Machida |
| Ebina General Hospital | Tomonori Kobayashi |
| Ehime Prefectural Central Hospital | Satoshi Fujiwara |
| Ehime University Hospital | Masahiko Tagawa |
| Fujieda Heisei Memorial Hospital | Kazuhiro Hashizume |
| Fujieda Municipal General Hospital | Yujiro Tanaka |
| Fujita General Hospital | Sato Masahiro |
| Fujita Health University Hospital | Ichiro Nakahara |
| Fujiyoshida Municipal Hospital | Syogo Imae |
| Fukaya　Red　Cross　Hospital | Toshiyuki Ohtani |
| Fukuchiyama City Hospital | Kei Owada |
| Fukui Red Cross Hospital | Masaki Nishimura |
| Fukuiken Saiseikai Hospital | Yasushi Takabatake |
| Fukuoka Neurosurgical Hospital | Hiroshi Aikawa |
| Fukuoka Seisyukai Hospital | Masaharu Tani |
| Fukuoka Shin Mizumaki Hospital | Kin Sigenari |
| Fukuoka University Chikushi Hospital | Toshio Higashi |
| Fukuoka University Hospital | Hiroshi Abe |
| Fukuokaken Saiseikai Futsukaichi Hospital | Yasuharu Takeuchi |
| Fukushima Medical University Hospital | Masazumi Fujii |
| Funabashi Municipal Medical Center | Hiromichi Naito |
| Gamagori City Hospital | Kazuo Koide |
| Gifu Municipal Hospital | Tetsuya Tanigawara |
| Gifu University Hospital | Toru Iwama |
| Gohshi Hospital | Yoshiharu Oki |
| Hachinohe City Hospital | Kawamura Tsuyoshi |
| Hachisuga　Hospital | Satoshi Inoha |
| Hakodate Neurosurgical Hospital | Takaaki Yamazaki |
| Hakujyuji Hospital | Shuji Hayashi |
| Hamamatsu University School of Medicine University Hospital | Hiroaki Neki |
| Handa City Hospital | Shinji Shimato |
| Harasanshin　Hospital | Tadahisa Shono |
| Hashima City Hospital | Hirotaka Watarai |
| Hata Kenmin Hospital | Yuji Nojima |
| Hibino　Hospital | Katsuzo Kiya |
| Hidaka Hospital | Shinji Obayashi |
| Higashiosaka City Medical Center | Takatoshi Fujimoto |
| Higashisumiyoshi Morimoto Hospital | Tadafumi Isono |
| Hiraka General Hospital | Susumu Fushimi |
| Hirosaki University Hospital | Atsushi Saito |
| Hiroshima Red Cross & Atomic-Bomb Survivors Hospital | Masayuki Sumida |
| Hiroshima University Hospital | Takahito Okazaki |
| Hokkaido Ohno Memorial Hospital | Taketo Kataoka |
| Hokkaido University Hospital | Toshiya Osanai |
| Hokushin General Hospital | Akihiro Tsukada |
| Hokuto Hospital | Kazumi Nitta |
| Hoshi General Hospital | Toru Kobayashi |
| Hyogo Prefectural Nishinomiya Hospital | Takayuki Sakaki |
| Ibaraki Seinan Medical Center Hospital | Keishi Fujita |
| Ichikikushikino Association Neurosurgery Center | Koichi Ishimaru |
| Iida Municipal Hospital | Sumio Kobayashi |
| Iizuka Hospital | Takahara Kenta |
| Ikeda Hospital | Kohsho Fujikawa |
| Imamura　General Hospital | Naoaki Kanda |
| Imari Arita Kyouritsu Hospital | Hirofumi Goto |
| Institute of Brain and Blood Vessels Mihara Memorial Hospital | Kazuma Kowata |
| Iseikai Hospital | Katsumi Matsumoto |
| Ishikawa Prefectural Central Hospital | Naoyuki Uchiyama |
| Ishikiriseiki Hospital | Tsuyoshi Inoue |
| Ishinkai Yao General Hospital | Yoshitomo Uchiyama |
| Itabashi Chuo Medical Center | Shoko Atsuchi |
| Itami Kousei Neurosurgical Hospital | Noda Shinya |
| Itsukaichi Memorial Hospital | Toshiyuki Tsuboi |
| Iwate Medical University | Kuniaki Ogasawara |
| Iwate Prefectural Kuji Hospital | Kazuyuki Miura |
| Iwate Prefectural Ofunato Hospital | Taro Suzuki |
| Izumi City General Hospital | Hisashi Kubota |
| Izumi Regional Medical Center | Tomohisa Okada |
| JA Onomichi General Hospital | Masaru Abiko |
| JA Toride General Medical Center | Yosihisa Kawano |
| Japan Community Health Care Organization Chukyo Hospital | Kenichiro Fujishiro |
| Japan Community Health Care Organization　Fukui Katsuyama General Hospital | Tsuyoshi Nakajima |
| Japan Community Health Care Organization Kumamoto General Hospital | Kazunari Koga |
| Japan Community Health Care Organization Kyusyu Hospital | Naoyuki Imamoto |
| Japan Community Health Care Organization Tokyo Takanawa Hospital | Hirofumi Hiyama |
| Japanese Red Cross Aichi Medical Center Nagoya Daini Hospital | Yukio Seki |
| Japanese Red Cross Ashikaga Hospital | Shunsuke Shibao |
| Japanese Red Cross Date Hospital | Takeshi Matsuoka |
| Japanese Red Cross Fukuoka Hospital | Jiro Kitayama |
| Japanese Red Cross Fukushima Hospital | Tsuyoshi Ichikawa |
| Japanese Red Cross Hachinohe Hospital | Toshinari Misaki |
| Japanese Red Cross Hamamatsu Hospital | Keisuke Ito |
| Japanese Red Cross Ise Hospital | Fumitaka Miya |
| Japanese Red Cross Koga Hospital | Hidenori Yokota |
| Japanese　Red　Cross　Kumamoto　Hospital | Tadashi Terasaki |
| Japanese Red Cross Kyoto Daiichi Hospital | Keisuke Imai |
| Japanese Red Cross Maebashi Hospital | Ken Asakura |
| Japanese Red Cross Matsue Hospital | Hiroki Fukuda |
| Japanese Red Cross Morioka Hospital | Tsukasa Wada |
| Japanese Red Cross Narita Hospital | Michio Nakamura |
| Japanese Red Cross Omori Hospital | Hideki Arakawa |
| Japanese Red Cross Shizuoka Hospital | Noboru Imai |
| Japanese Red Cross Society Himeji Hospital | Kazuya Takahashi |
| Japanese Red Cross Society Iiyama Hospital | Kunihiko Kodama |
| Japanese Red Cross Society Kyoto Daini Hospital | Shogo Ogita |
| JCHO Kobe Central Hospital | Keigo Matsumoto |
| Jichi Medical University Hospital | Ryota Tanaka |
| Jisenkai Aizawa Hospital | Kazuo Kitazawa |
| Juntendo University Shizuoka Hospital | Takuji Yamamoto |
| Juntendo University Urayasu Hospital | Kazuo Yamashiro |
| Kaga Medical Center | Naoki Shirasaki |
| Kagawa University Hospital | Masanobu Okauchi |
| Kagoshima City Hospital | Hiroshi Tokimura |
| Kagoshima University Hospital | Shunichi Tanaka |
| Kaisei General Hospital | Koichi Okiya |
| Kakogawa Central City Hospital | Keiji Kidoguchi |
| Kameda Medical Center | Tetsuo Ando |
| Kanazawa Neurosurgical Hospital | Nobutaka Yamamoto |
| Kanbara Public Hospitals Association | Kazuyuki Nishigaya |
| Kaneda Hospital | Masayuki Mizobuchi |
| Kansai Electric Power Hospital | Morio Takasaki |
| Kansai Medical University Hospital | Kunikazu Yoshimura |
| Kariya Toyota General Hospital | Toshihisa Nishizawa |
| Kashiwaba Neurosurgical Hospital | Katsuhiko Maruichi |
| Kawachi General Hospital | Kazutami Nakao |
| Kawasaki Hospital | Junya Hayashi |
| Kawasaki Hospital | Hiroshi Hayashi |
| Kazuno Kousei Hospital | Masayuki Sasou |
| Keishunkai Medical Corporation Kobari General Hospital | Naoaki Sato |
| Kenoutokorozawa Hospital | Hideaki Ishihara |
| Kimitsu Chuo Hospital | Michihiro Hayasaka |
| Kin-ikyo Chuo Hospital | Ryuhei Kouno |
| Kiryu Kosei General Hospital | Satoshi Magarisawa |
| Kishiwada City Hospital | Osamu Kawakami |
| Kishiwada Tokushukai Hospital | Hiroyuki Matumoto |
| Kitaharima Medical Center | Shigeru Miyake |
| Kitakyushu General Hospital | Masaru Idei |
| Kitakyushu Municipal Medical Center | Tsukamoto Haruhisa |
| Kitakyusyu City Yahata Hospital | Ryo Miyaoka |
| Kitami Red Cross Hospital | Teruo Kimura |
| Kitamurayama Hospital | Kenta Kunimoto |
| Kitasato University Hospital | Toshihiro Kumabe |
| Kobe City Medical Center General Hospital | Nobuyuki Sakai |
| Kobe City Nishi-Kobe Medical Center | Noriaki Ashida |
| Kobe Ekisaikai Hospital | Takashi Tominaga |
| Kobe University Hospital | Atsushi Fujita |
| Kochi Health Sciences Center | Hiroyuki Nishimura |
| Kochi Medical School Hospital | Hitoshi Fukuda |
| Kofu Neurosurgical Hospital | Toyoaki Shinohara |
| Kohnan Hospital | Yukako Yazawa |
| Kohnan Medical Center | Yasuhiko Motooka |
| Kohsei General Hospital | Kenjiro Fujiwara |
| Kokura Memorial Hospital | Taketo Hatano |
| Komaki City Hospital | Takenori Kato |
| Komatsu Municipal Hospital | Yuuichi Hirota |
| Koseiren Tsurumi Hospital | Akihiko Kaga |
| Kouseiren Takaoka Hospital | Masayuki Arai |
| Koyama Memorial Hospital | Takuya Kawai |
| Kumamoto University Hospital | Yasuyuki Kaku |
| Kurashiki Central Hospital | Masaki Chin |
| Kurobe City Hospital | Hiromichi Yamamoto |
| Kuroishi General Hospital | Kosuke Katayama |
| Kurosawa Hospital | Takeshi Ogura |
| Kurume University Hospital | Motohiro Morioka |
| Kushiro Rosai Hospital | Daisuke Shimbo |
| Kuwana City Medical Center | Hiroshi Sakaida |
| Kyoritsu Hospital of Kyowakai Medical Corporation | Masayuki Yokota |
| Kyoto Kizugawa Hospital | Tatsuhito Yamagami |
| Kyoto Prefectural University of Medicine | Tomoyuki Ohara |
| Kyoto Tanabe Central Hospital | Mamoru Murakami |
| Kyoto-Katsura Hospital | Norio Nakajima |
| Kyowa Chuo Hospital | Norikazu Kurokawa |
| Kyushu Central Hospital of The Mutual Aid Association of Public School Teachers | Seiji Gotoh |
| Kyushu Rosai Hospital | Sei Haga |
| Kyushu University Hospital | Koji Yoshimoto |
| Makita General Hospital | Yoshinori Arai |
| Maruko Central Hospital | Toshiyuki Tsukada |
| Maruyama Memorial General Hospital | Shigeyoshi Kimura |
| Masu Memorial Hospital | Mamoru Ota |
| Masuda Red Cross Hospital | Matsui Ryukichi |
| Matsubara Tokushukai Hospital | Kenji Ooyama |
| Matsuyama-Shimin-Hsp | Yuji Yamamoto |
| Mazda Hospital | Yukihiko Kawamoto |
| Medical Corporation Tokushukai Ogakitokushukai Hospital | Katsuhiko Hayasi |
| Meijibashi Hospital | Toshikazu Takeshima |
| Meitetsu Hospital | Yotaro　Takeuchi |
| Midorigaoka Hospital | Arai Motohiro |
| Mie Chuo Medical Center National Hospital Organization | Fujimaro Ishida |
| Mie University Hospital | Hidenori Suzuki |
| Minaminagano Medical Center Shinonoi General Hospital | Takahiro Murata |
| Mito Brain Heart Center | Yoichi Harada |
| Mitsugi Public General Hospital | Takashi Matsuoka |
| Miyazaki Prefectural Nobeoka Hospital | Megumi　Nitta |
| Miyoshi Central Hospital | Osamu Hamasaki |
| Mominoki Hospital | Masanori Morimoto |
| Munakata Suikokai General Hospital | Yoshimasa Kinoshita |
| Municipal Tsuruga Hospital | Yosikazu Arai |
| Murata Hospital | Akihiro Itoh |
| Muroran City General Hospital | Hiroshi Ooyama |
| Nadogaya Hospital | Yasuaki Inoue |
| Nagahama City Hospital | Satoshi Horiguchi |
| Nagano Municipal Hospital | Yosikazu Kusano |
| Nagasaki Rosai Hospital | Makoto Hirose |
| Nagaski University Hospital | Tsuyoshi Izumo |
| Nagoya Tokushukai General Hospital | Amano Takayuki |
| Nakatsu Municipal Hospital | Hiromichi Koga |
| Nara City Hospital | Hidemori Tokunaga |
| Nara Prefecture General Medical Center | Kenta Fujimoto |
| Nasu Red Cross Hospital | Akiko Arakawa |
| National Cerebral and Cardiovascular Center | Masatoshi Koga |
| National Hospital Organization Beppu Medical Center | Yu Takeda |
| National Hospital Organization Hamada Medical Center | Yoroyoshi Kimura |
| National Hospital Organization　Higashihiroshima　Medical　Center | Takashi Sadatomo |
| National Hospital Organization Iwakuni Clinical Center | Kotaro Ogihara |
| National Hospital Organization Kanmon Medical Center | Katsuhiro Yamashita |
| National Hospital Organization Kumamoto Medical Center | Takashi Nakagawa |
| National Hospital Organization Kyoto Medical Center | Shunichi Fukuda |
| National Hospital Organization Okayama Medical Center | Yasuhiro Manabe |
| Nayoro City General Hospital | Naoki Tokumitsu |
| New Tokyo Hospital | Norihiro Ishii |
| NHO Sendai Medical Center | Masayuki Ezura |
| NHO Toyohashi Medical Center | Hideki Sakai |
| NHO Ureshino Medical Center | Miho Higashi |
| Nihon University Itabashi Hospital | Atsuo Yoshino |
| Niigata City General Hospital | Ken-Ichi Morita |
| Niigata Neurosurgical Hospital | Kiyoshi Onda |
| Nipponbashi Hospital | Shunichi Yoneda |
| Nishichita General Hospital | Shigeta Moriya |
| Nishijima Hospital | Masaki Sakamoto |
| Nishinomiya Kyoritsu Neurosurgical Hospital | Masao Tsuji |
| Nishiwaki Municipal Hospital | Shigenori Katayama |
| Noto General Hospital | Yasuhiro Aida |
| Numata Neurosurgery and Heart Disease Hospital | Norihiko Akao |
| Obase Hospital | Haruki Takahashi |
| Obihiro Kosei Hospital | Masafumi Ohtaki |
| Odate Municipal General Hospital | Naoya Shimada |
| Ohkawara Neurosurgical Hospital | Takahiro Maeda |
| Ohnishi Neurological Hospital | Yoshihiro Kuga |
| Ohta Nishinouchi Hospital | Masahisa Kawakami |
| Oita Prefectural Hospital | Yasuyuki Nagai |
| Okanami General Hospital | Keita Suzuki |
| Okayama City Hospital | Koji Tokunaga |
| Okayama Kyokuto Hospital | Akira Handa |
| Okayama University Hospital | Isao Date |
| Okinawa Kyodo Hospital | Koji Idomari |
| Okinawa Prefectural Nanbu Medical Center &Children's Medical Center | Tomoaki Nagamine |
| Okinawa Prefectural Yaeyama Hospital | Shinobu Adachi |
| Omachi Municipal General Hospital | Toshiki Aoki |
| Omihachiman Community Medical Center | Masayuki Nakajima |
| Omuta City Hospital | Terukazu Kuramoto |
| Oozu Central Hospital | Nishihara Jun |
| Osaka City General Hospital | Tomoya Ishiguro |
| Osaka General Medical Center | Manabu Sakaguchi |
| Osaka Neurological Institute | Akatsuki Wakayama |
| Osaka Neurosurgical Hospital | Naohiro Osaka |
| Osaka Rosai Hospital | Hiroyuki Hashimoto |
| Osaki Citizen Hospital | Masahiro Yoshida |
| Red Cross Osaka Hospital | Kenji Hashimoto |
| Saga-Ken Medical Centre Koseikan | Kenichi Matsumoto |
| Saiseikai Ibaraki Hospital | Yasunobu Goto |
| Saiseikai Imabari Hospital | Tatunori Kawai |
| Saiseikai Karatsu Hospital | Toshiro Katsuta |
| Saiseikai Kawaguchi General Hospital | Seisuke Iseki |
| Saiseikai Kumamoto Hospital | Toshiro Yonehara |
| Saiseikai Matsusaka General Hospital | Hiroto Murata |
| Saiseikai Toyama Hospital | Michiya Kubo |
| Saiseikai Utsunomiya Hospital | Masashi Nakatsukasa |
| Saiseikai Yahata General Hospital | Yuuzi Okamoto |
| Saiseikai Yamagatasaisei Hospital | Sunao Takemura |
| Saiseikai Yokohamashi Tobu Hospital | Makoto Inaba |
| Saitama City Hospital | Atsuhiro Kojima |
| Saitama Medical Center | Soichi Oya |
| Saitama Medical University International Medical Center | Shinya Kohyama |
| Saitama　Prefectural　Cardiovascular and Respiratory Center | Yuichiro Kikkawa |
| Saitama Red Cross Hospital | Toshie Takahashi |
| Sakai City Medical Center | Takashi Tsuzuki |
| Saku Central Hospital Advanced Care Center | Takaaki Yoshida |
| Sannocho Hospital | Tsunenori Ozawa |
| Sanyudo Hospital | Youhei Kudoh |
| Sapporo City General Hospital | Takigami Masayoshi |
| Sapporo Medical University School of Medicine | Nobuhiro Mikuni |
| Sapporo Shiroishi Memorial Hospital | Ken Takahashi |
| Sapporo Teishinkai Hospital | Tanikawa Rokuya |
| Sasebo Chuo Hospital | Kouichirou Takemoto |
| Satodaiichi Hospital | Shigehiro Nakahara |
| Seirei Hamamatsu General Hospital | Kazunari Homma |
| Seirei Memorial Hospital | Akiyoshi Sato |
| Seirei Mikatahara General Hospital | Soichi Akamine |
| Sendai East Neurosurgical Hospital | Watabe Noriaki |
| Shiga University of Medical Science | Kazuhiko Nozaki |
| Shikoku Medical Center for Children and Adults | Shinya Okita |
| Shimizu-Hospital | Takashi Yoshida |
| Shin Koga Hospital | Tsutomu Hitotsumatsu |
| Shin-Yurigaoka General Hospital | Jinichi Sasanuma |
| Shinshu University Hospital | Yoshiki Hanaoka |
| Shinsuma Hospital | Takashi Mizowaki |
| Shintakeo Hospital | Makoto Ichinose |
| Shinyukuhashi Hospital | Shingo Yamashita |
| Shiroyama Hospital | Hiroshi Shimano |
| Shizuoka City Shizuoka Hospital | Seiji Fukazawa |
| Showa Inan General Hospital | Shinsuke Muraoka |
| Shunan Memorial Hospital | Masaru Honda |
| Shunan Municiple Sinnanyo City Hospital | Yasuhiro Fujii |
| Social Medical Corporation Ijinkai Nakamura Hospital | Kenji Kamiyama |
| Social Medical Corporation Kotobukikai Tominaga Hospital | Masahiko Kitano |
| Sonoda Daiichi Hospital | Kentaro Shimoda |
| Soseikai General Hospital | Noriaki Matsubara |
| South Miyagi Medical Center | Takashi Inoue |
| Southern Tohoku General Hospital | Zenitirou Watanabe |
| Southern Tohoku General Hospital | Shinjitsu Nishimura |
| St. Mary's Hospital | Yoshihisa Fukushima |
| Suiseikai Kajikawa Hospital | Eiji Imamura |
| Suwa Red Cross Hospital | Naomichi Wada |
| Suzuka Chuo General Hospital | Shigetoshi Shimizu |
| Suzuka Kaisei Hospital | Tomohiro Araki |
| Tachikawa General Hospital | Hiroshi Abe |
| Takai Hospital | Tetuya Morimoto |
| Takamatsu Municipal Hospital | Kenji Shono |
| Takamatsu Red Cross Hospital | Masahiro Kagawa |
| Takeda Healthcare Foundation Takeda General Hospital | Kazuhiko Nishino |
| Takeda Hospital | Nobutake Sadamasa |
| Tane General Hospital | Shoichi Shiraishi |
| Tanegashima Medical Center | Soichiro Komasaku |
| Teikyo University Hospital | Masaaki Shojima |
| Teine Keijinkai Hospital | Katsuyuki Asaoka |
| The Jikei University Hospital | Tohru Sano |
| The University of Tokyo Hospital | Hiroki Hongo |
| TMG Asaka Medical Center | Hidetoshi Nakamoto |
| Tobata Kyoritsu Hospital | Takehisa Tsuji |
| Toho University Ohashi Medical Center | Morito Hayashi |
| Tohoku Medical and Pharmaceutical University Hospital | Tatsuya Sasaki |
| Tokai Central Hospital | Jun Yamada |
| Toki Municipal General Hospital | Hideomi Kitajima |
| Tokorozawa Chuo Hospital | Hiroshi Miyazaki |
| Tokuda Neurosurgical Hospital | Akihito Hashiguchi |
| Tokushima Municipal Hospital | Hiromi Ueta |
| Tokushima Prefecture Naruto Hospital | Masahito Agawa |
| Tokushima University Graduate School of Biomedical Sciences | Yasushi Takagi |
| Tokuyama Central Hospital | Harada Kunihiko |
| Tokyo Bay Urayasu Ichikawa Medical Center | Kunisasu Saigusa |
| Tokyo Dental College Ichikawa General Hospital | Masateru Katayama |
| Tokyo Medical and Dental University | Taketoshi Maehara |
| Tokyo Metropolitan Hiroo Hospital | Kensaku Yoshida |
| Tokyo Rosai Hospital | Koichi Kato |
| Tokyo Saiseikai Central Hospital | Haruhiko Hoshino |
| Tokyo Women’s Medical University | Tatsuya Ishikawa |
| Tokyo Women’s Medical University Yachiyo Medical Center | Akitsugu Kawashima |
| Tomakomai City Hospital | Tomohiro Yamauchi |
| Tonami General Hospital | Toru Masuoka |
| Tottori City Hospital | Keiichi Akatsuka |
| Tottori Seikyo Hospital | Motoi Saito |
| Tottori University Hospital | Makoto Sakamoto |
| Toumei Atsugi Hospital | Keiichiro Onitsuka |
| Toyama City Hospital | Masanao Mohri |
| Toyota Memorial Hospital | Oheda Motoki |
| Tsuchiura Kyodo General Hospital | Akira Machida |
| Tsukazaki Hospital | Yoshihiko Fu |
| Tsukuba Medical Center Hospital | Masahiko Hiroki |
| Tsukuba Memorial Hospital | Hitoshi Aiyama |
| Tsushima City Hospital | Tomoko Yamana |
| Tsuyama Chuo Hospital | Kazuki Kobayashi |
| Ube Industries Central Hospital | Norio Ikeda |
| Uji-Tokushukai Hospital | Nozomu Murai |
| University of Miyazaki Hospital | Hajime Oota |
| University of Occupational and Environmental Health Japan | Yuko Tanaka |
| University of Toyama | Satoshi Kuroda |
| University of Tsukuba Hospital | Mikito Hayakawa |
| Utsunomiya Memorial Hospital | Norihiro Sito |
| Wakayama Medical University | Naoyuki Nakao |
| Yagi Neurosurgical Hospital | Hirokatsu Taniguchi |
| Yaizu City Hospital | Miyuki Ishikawa |
| Yamagata University Hospital | Sonoda Yukihiko |
| Yamaguchi Prefectural Grand Medical Center | Yuka Itou |
| Yamaguchi Red Cross Hospital | Yasuhiro Hamada |
| Yamaguchi University Hospital | Ishihara Hideyuki |
| Yamakoukai Ueyama Hospital | Takaaki Inomoto |
| Yamamoto Third Hospital | Yasunobu Mita |
| Yamanashi Kosei Hospital | Mikito Uchida |
| Yamanashi Prefectural Central Hospital | Shin Nakano |
| Yamanashi Red Cross Hospital | Hiroshi Ozawa |
| Yame General Hospital | Takahiro Miyahara |
| Yatsu Hoken Hospital | Naoyuki Sakai |
| Yodogawa Christian Hospital | Masashi Morikawa |
| Yokohama Brain and Spine Center | Yasunobu Nakai |
| Yokohama City University Hospital | Nobuyuki Shimizu |
| Yokohama City University Medical Center | Katsumi Sakata |
| Yokohama General Hospital | Masanori Nakagawa |
| Yokohama Rosai Hospial | Ichiro Imafuku |
| Yokohamashintoshi Neurosurgical Hospital | Mitsuhiro Iwasaki |
| Yokosuka Kyosai Hospital | Takashi Irioka |
| Yoshida Hospital.Cerebrovascular Research Institute | Yasuhisa Yoshida |
| Yuaikai Hospital | Kinya Nakanishi |
| Yuri Kumiai General Hospital | Yoshitaka Suda |
